# Proteasome system dysregulation and treatment resistance mechanisms in major depressive disorder

**DOI:** 10.1038/tp.2015.180

**Published:** 2015-12-01

**Authors:** A Minelli, C Magri, A Barbon, C Bonvicini, M Segala, C Congiu, S Bignotti, E Milanesi, L Trabucchi, N Cattane, M Bortolomasi, M Gennarelli

**Affiliations:** 1 Department of Molecular and Translational Medicine, Biology and Genetic Division, University of Brescia, Brescia, Italy; 2 Genetic Unit, IRCCS Istituto Centro San Giovanni di Dio Fatebenefratelli, Brescia, Italy; 3 Psychiatric Hospital ‘Villa Santa Chiara', Verona, Italy; 4 Psychiatric Unit, IRCCS Istituto Centro San Giovanni di Dio Fatebenefratelli, Brescia, Italy; 5 Department of Human Molecular Genetics and Biochemistry, Sackler Faculty of Medicine, Tel Aviv University, Tel Aviv, Israel

## Abstract

Several studies have demonstrated that allelic variants related to inflammation and the immune system may increase the risk for major depressive disorder (MDD) and reduce patient responsiveness to antidepressant treatment. Proteasomes are fundamental complexes that contribute to the regulation of T-cell function. Only one study has shown a putative role of proteasomal *PSMA7*, *PSMD9* and *PSMD13* genes in the susceptibility to an antidepressant response, and sparse data are available regarding the potential alterations in proteasome expression in psychiatric disorders such as MDD. The aim of this study was to clarify the role of these genes in the mechanisms underlying the response/resistance to MDD treatment. We performed a case-control association study on 621 MDD patients, of whom 390 were classified as treatment-resistant depression (TRD), and we collected peripheral blood cells and fibroblasts for mRNA expression analyses. The analyses showed that subjects carrying the homozygous GG genotype of *PSMD13* rs3817629 had a twofold greater risk of developing TRD and exhibited a lower *PSMD13* mRNA level in fibroblasts than subjects carrying the A allele. In addition, we found a positive association between *PSMD9* rs1043307 and the presence of anxiety disorders in comorbidity with MDD, although this result was not significant following correction for multiple comparisons. In conclusion, by confirming the involvement of *PSMD13* in the MDD treatment response, our data corroborate the hypothesis that the dysregulation of the complex responsible for the degradation of intracellular proteins and potentially controlling autoimmunity- and immune tolerance–related processes may be involved in several phenotypes, including the TRD.

## Introduction

Major depressive disorder (MDD) is a major health problem associated with gradual and often incomplete recovery and with significant limitations in functioning and well-being. Unfortunately, several studies have shown that only about one-third of patients experience full remission after first-choice treatment, and that for many patients achieving remission requires repeated trials of sufficiently dosed antidepressant medication.^1, 2^ In addition, 15–30% of MDD patients are classified as having treatment-resistant depression (TRD).^3, 4^

Carvalho *et al.*^5, 6^ reported that increased activation of the inflammatory system is concordant with hyperactivity of the hypothalamic–pituitary–adrenal axis and with impaired glucocorticoid receptor (GR) sensitivity in TRD patients. These findings were corroborated by genetics studies indicating that functional allelic variants in the interleukin (IL)-1beta (IL-1b), tumor necrosis factor-α and C-reactive protein genes, as well as genetic variations affecting T-cell function, may increase the risk for depression. Moreover, single nucleotide polymorphisms (SNPs) in the IL-1b, IL-6 and IL-11 genes and in those regulating T-cell function may be associated with reduced responsiveness to antidepressant therapy.^7^

Although the aforementioned ILs and other factors have been extensively studied in MDD and in the response to its treatment, few studies of T-cell function are available to date.

Fundamental complexes that contribute to regulate T-cell function are proteasomes. In fact, proteasomes are the major multi-catalytic enzyme complexes involved in the intracellular degradation of ubiquitinated proteins and in the production of small protein fragments that can be used by major histocompatibility complex class I to present antigens to the immune system. Protein degradation is fundamental for many important biological functions, such as immune surveillance, cell cycle progression, apoptosis and synaptic reorganization; therefore, the disruption or alteration of this mechanism due to genetic variants can have a relevant impact on several diseases.^8^

Based on genetic studies, the ubiquitin–proteasome system has been identified as a canonical pathway that is associated with neuropsychiatric disorders such as Alzheimer disease,^9^ psychosis^10^ and bipolar disorder.^11, 12, 13^ In particular, two studies showed that several proteasome 26S subunits are significantly downregulated in schizophrenia,^14, 15^ and Bousman *et al.*^11^ suggested that the dysregulation of the ubiquitin–proteasome system may partially determine the manifestation and the severity of psychotic symptoms in schizophrenia and mood disorders.

Only one study has shown a putative role of proteasomal subunit genes in the susceptibility to MDD and the antidepressant response.^16^ The authors observed that the proteasome subunit β4 (*PSMD4*) gene is involved in etiopathogenesis of MDD, although this result was not replicated in the largest genome-wide association study on MDD.^17^ Alternatively, Wong *et al.*^16^ found significant associations of the treatment response with the 26S proteasome non-ATPase subunit 9 (*PSMD9*), proteasome alpha type 7 subunit (*PSMA7*) and *PSMD13* genes. Recently, the *PSMD9* gene has been found to be associated with generalized anxiety disorder.^18^

Finally, although data are available regarding the proteasome expression levels in human peripheral tissues in MDD patients,^19, 20^ to the best of our knowledge, no data have been reported concerning the relationship between the expression pattern of the ubiquitin–proteasome system and the antidepressant treatment response. Peripheral blood cells (PBCs) are frequently used to identify depression biomarkers;^21^ however, recent evidence has indicated that fibroblasts represent a promising model for studying several processes related to MDD^22^ because many possible confounding factors that are often associated with patient PBCs, such as diet, lifestyle, smoking status and drug treatment, may be virtually eliminated after several cycles of cell division.^23^

Based on the above findings, to better understand the role of proteasome genes in the mechanisms underlying the MDD treatment response, we (1) investigated the potential roles of three genes (*PSMA7*, *PSMD9* and *PSMD13*) previously found to be associated with drug responses,^16^ (2) performed single-gene re-analysis on the Sequenced Treatment Alternatives to Relieve Depression (STAR*D) data set and (3) analyzed the expression profiles of PBCs and fibroblasts.

## Materials and methods

### Sample

About 621 MDD patients with at least moderate to severe depression, who met Diagnostic and Statistical Manual of Mental Disorders-IV (DSM-IV) classification system criteria were voluntarily enrolled in the study. All of them had been referred to the Villa S. Chiara Psychiatric Hospital in Verona or to the Psychiatry Rehabilitation Unit of IRCCS Centro Fatebenefratelli ‘S. Giovanni di Dio' in Brescia.

Diagnosis of unipolar depression was confirmed using the Structured Clinical Interview for DSM-IV Axis I Disorders (SCID-I) diagnostic scale. The exclusion criteria were as follows: (a) mental retardation or cognitive disorder; (b) a lifetime history of schizophrenic, schizoaffective or bipolar disorder; (c) personality disorder, substance abuse, alcohol abuse or dependency, obsessive-compulsive disorder or post-traumatic stress disorder as the primary diagnosis; and (d) comorbidity with an eating disorder.

About 147 patients (23.7%) showed psychotic symptoms; 258 (41.5%) showed current comorbidity in Axis I (generalized anxiety disorder, panic attacks or anxiety disorder not otherwise specified), 139 (22.4%) showed symptoms of Axis II disorders (paranoid, dependent, obsessive-compulsive or histrionic personality disorder) and 24 (3.9%) alcohol abuse, as a secondary diagnosis (the total number exceeded the number of subjects due to the presence of comorbidities).

About 390 subjects were classified as TRD patients (Group 1: TRD). Treatment resistance to antidepressants was defined as the failure to respond to two or more adequate trials of two or more different classes of antidepressants and to an adequate trial of a tricyclic antidepressant drug; such patients were classified as Stage III according to the Thase and Rush staging method.^24^

The second group was composed of 231 MDD patients experiencing a depressive episode who responded to their current treatment (Group 2: non-TRD).

The control sample consisted of 467 unrelated healthy volunteers who were screened for DSM-IV Axis I disorders by expert psychologists using the Mini-International Neuropsychiatric Interview (M.I.N.I.).^25^ Only healthy volunteers without a history of drug or alcohol abuse or dependence and without a personal or first-degree family history of psychiatric disorders were enrolled in the study. Furthermore, subjects who obtained a score <27/30 on the Mini Mental State Examination (M.M.S.E.)^26^ were excluded from the study.

All socio-demographic and clinical characteristics for all groups are shown in Table 1.

We performed transcriptional analyses on 59 PBCs (from 37 MDD patients, including 20 non-TRD and 17 TRD patients, and 22 controls) and 48 fibroblasts samples (from 27 MDD patients, including 17 non-TRD and 10 TRD patients, and 21 controls). The socio-demographic and clinical features for both tissue samples are shown in Table 2.

Only for transcriptional analyses, we used the following additional exclusion criteria: (1) age >70 years old, because fibroblasts have a shorter life span in culture; (2) presence of metabolic disorders (for example, diabetes); and (3) presence of specific skin diseases (for example, skin cancer or psoriasis).

Both patients and controls were Caucasians of Italian descent for at least two generations, residing in north Italy and unrelated to other participants.

The study was approved by the Local Ethics Committees (CEIOC IRCCS Istituto Centro San Giovanni di Dio Fatebenefratelli, Brescia N: 50/2008 and Ethics Committee of the province of Verona N: 4997/09.11.01), and written informed consent was obtained.

### Genotyping

*PSMD13* rs3817629, *PSMD9* rs1043307 and *PSMA7* rs2057168 were genotyped using the BeadXpress System and the VeraCode Assay according to the manufacturer's instructions (http://www.illumina.com). The raw BeadXpress data were processed using the Illumina BeadStudio software suite (genotyping module 3.3.7; San Diego, CA, USA), producing report files containing normalized intensity data and SNP genotypes.

The genotyping of *PSMD13* rs3817629 and *PSMD9* rs1043307 was repeated via the SNaPshot assay as a quality control. All details regarding the SNaPshot assay are available on request.

### LD analysis

For the intronic SNP *PSMD13* rs3817629, that was positively associated with response, we tested for linkage disequilibrium (LD) with other variants selected for their putative effects on protein function. Using the 1000 Genome database data, we computed the LD coefficient (*r*^2^) between the *PSMD13* rs3817629 and all SNPs mapping inside *PSMD13* gene and 10 kb downstream and upstream the gene. Then we selected those displaying *r*^2^>0.8. For coding variants, functional prediction was performed using the Variant Annotation Integrator (http://genome.ucsc.edu/index.html), and for noncoding variants, functional prediction was performed using the Regulome database (http://regulomedb.org/).^27^

### STAR*D data sets replication study

We evaluated genetic data from data set 2 and 3 of the STAR*D study^28^ (https://www.nimhgenetics.org/) – distribution 3.02, selecting only patients defined as ‘White' we categorized these patients into the non-TRD and TRD groups. We defined any MDD patient who did not respond to two or more trials of different classes of antidepressants as TRD. We excluded subjects who responded to citalopram at Level 1 of the study from the non-TRD group to avoid placebo effects.^29, 30^ The population consisted of 259 non-TRD and 186 TRD patients. Logistic regression analyses were performed to evaluate differences in the genotype frequency distribution between the two groups. To correct for ethnic heterogeneity, we performed principal component analysis, and we used the first three components as covariates in the regression analyses. None of the analyzed SNPs was directly genotyped in the STAR*D cohort. Therefore, for the SNP *PSMA7* rs2057168, the association was indirectly tested by analyzing the SNP rs1535669, which displayed high LD (*r*^2^=0.95) with rs2057168 in the European population accordingly to the NetAffx database of Affymetrix (http://www.affymetrix.com). For *PSMD13* rs3817629 and *PSMD9* rs1043307, because no SNPs displaying LD were available in the STARD data set, the association was tested using dosage data of the imputed genotypes. The SNPs within chromosomal regions (Hg19) chr11:236,808–252,984 and chr^2^0:122,326,812–122,386,604 were imputed using Beagle software^31^ and using the 1000 Genome database.

All logistic regression analyses were performed using Plink software (v.1.9).^32, 33^

### Cell culture and RNA isolation

Skin biopsies (3 mm^2^) were collected under local anesthesia from the scapular region under aseptic conditions and were immediately immersed in saline solution (phosphate-buffered saline). All primary cultures were grown in Eagle's minimum essential medium (Invitrogen-Life Technologies, Carlsbad, CA, USA) supplemented with fetal bovine serum (10%), penicillin (100 U ml^−1^), streptomycin (100 μg ml^−1^), non-essential amino acids (1% v/v) and glutamine (1% v/v) under optimal conditions (37 °C and 5% CO_2_). The medium was replaced every 3 days. When the fibroblasts reached confluence, the cells were subcultured into larger tissue culture dishes or were frozen in 20% foetal bovine serum and 10% DMSO. All fibroblasts were cultured until the fifth passage. Fibroblast cell cultures were screened for mycoplasma infection and mycoplasma removal agents were used to avoid and prevent the risk of contamination.

RNA was extracted from fibroblasts using the NucleoSpin kit (Carlo Erba Reagenti, Milan, Italy) according to the manufacturer's instructions.

Blood samples were collected between 0800 and 0900 hours after an overnight fast via venepuncture using PaxGene Tubes (Qiagen, Crawley, UK). RNA isolation was performed using the PaxGene Blood RNA Kit (Qiagen) according to the manufacturer's instructions.

One microgram of total RNA was used for complementary DNA synthesis using random hexamer primers (Invitrogen-Life Technologies) and Superscript II Reverse Transcriptase (Invitrogen-Life Technologies) according to the manufacturer's instructions.

### Real-time PCR analyses

For those genes in which a positive genotype association was found (*PSMD13* and *PSMD9*), the mRNA expression level was evaluated in both fibroblasts and PBCs.

The transcriptional expression levels were determined with TaqMan assays (Applied Biosystems, Foster City, CA, USA) performed on a StepOnePlus instrument (Applied Biosystems) according to the manufacturer's protocol.

In a pilot study in fibroblasts, we found that GAPDH is among the most stable reference genes in this tissue (data not shown). Moreover, GAPDH has been already used as a reference in MDD studies using fibroblasts and PBCs.^34, 35, 36^ Therefore, the relative expression levels of the target genes in the patient groups were calculated according to the comparative Ct method (^−ΔΔCt^ method)^37^ using GAPDH as the reference gene. Each determination was repeated in duplicate.

### Statistical analyses

Analysis of variance was used to detect possible differences in age and education between controls and MDD, as well as between TRD and non-TRD. *χ*^2^-tests were used to detect differences in demographic and clinical variables between the groups (Table 1). The differences in genotype frequency distribution for the three SNPs between MDD and controls were tested using a logistic regression analysis. Since the two groups differed in terms of gender, age and education (see Table 1) these variables were considered as covariates in the model. The same analyses were performed to test differences in genotype frequency distribution between TRD and non-TRD, and education (Table 1) was considered as covariate in the model. Moreover, we performed secondary analyses by logistic regression to investigate the putative influences of these three SNPs on comorbidity with anxiety disorders and the presence of psychotic symptoms. The additive, dominant and recessive genotype models were tested. All *P*-values were adjusted applying Bonferroni correction for all comparisons (*N=*36). Odds ratios and corresponding 95% confidence intervals (CIs) were used to quantify the association. All the genotype analyses were performed with Plink (v. 1.9) software.

According to G*Power Calculator software^38, 39^ in our cohorts of TRD e non-TRD patients, we have a power greater than 95% (for *α*<0.0014) of detecting the same allele differences (odds ratios<0.44), reported in the study by Wong *et al.*^16^ between responder and nonresponder.

The Kruskal–Wallis and Mann–Whitney *U* non-parametric tests were used to evaluate differences in *PSMD13* and *PSMD9* mRNA levels between MDD and controls, as well as between TRD and non-TRD, and to evaluate the association between the expression levels of the same genes with the relative polymorphisms. Pearson's coefficient was used to evaluate bivariate correlations between mRNA expression levels and the socio-demographic and clinical variables reported in Table 2.

Parametric and non-parametric tests were used meeting relative assumptions (for example, distribution, sample size).

All statistical analyses were conducted using SPSS version 17.0 (SPSS, Chicago, IL, USA).

## Results

### Genetic analysis results for our sample

As reported in Table 3, none of the analyzed SNPs showed a significantly different genotype frequency distribution between the controls and the patients using the additive model. The identical results were observed using the dominant and recessive models (data not reported).

When the patients were stratified into the TRD and non-TRD groups, differences in the genotype distribution of *PSMD13* rs3817629 was observed (*P=*0.004); GG homozygotes were more frequent in TRD patient group (56%, *P=*0.00045, OR=1.75, 95% CI: 1.26–2.45) (Table 4). This difference remained significant after adjustment for multiple comparisons (*P=*0.016).

The Regulome database reported that *PSMD13* rs3817629 is an intronic SNP displaying minimal evidence of affecting transcription factor-binding sites (score: 4). LD analysis of the genotypic data from the 1000 Genome database revealed that *PSMD13* rs3817629 is in high LD with 40 SNPs located inside or close to *PSMD13* gene, 6 of which are missense variants mapping to the first or second exon of the *PSMD13* gene and predicted as benign using Polyphen (http://genetics.bwh.harvard.edu/pph/) (Supplementary Figure 1S). Moreover, rs10902110 and rs10794302, were identified as intronic variants expected to affect the binding of transcription factors (Regulome score: 2b). Chromatin immunoprecipitation sequencing (chip-seq) analysis indicated that rs10902110, which maps to the first intron of the *PSMD13* gene, is located in an open chromatin locus that binds to RNA polymerase II and other transcription factors such as CREBBP, RFX3, MYC, SP1, GABPA and ELF1^40, 41, 42^ (ENCODE project). Alternatively, rs10794302 maps to the first intron of the *SIRT3* gene at a locus predicted to have an open chromatin structure and a transcription factor-binding motif. Chip-seq analysis suggested that the GR (NR3C1) binds to this region (ENCODE project). Significant associations were detected between the presence of anxiety disorders in comorbidity with MDD and *PSMD9* rs1043307. The genotype frequency distribution of this SNP was significantly different between MDD patients with and without anxiety disorders according to the additive model (*P=*0.035). In particular, subjects carrying the AA or AG genotype exhibited a 1.76-fold (95% CI 0.98–3.18) and 1.38-fold (95% CI 0.76–2.51) increased risk of anxiety comorbidity relative to subjects carrying the homozygous GG genotype, respectively. However, this result did not remain significant after adjustment for multiple comparisons.

### Replication of the genotypic data in the STAR*D cohort

To confirm our results, we replicated the genotypic analyses in subgroups of STAR*D patients. As shown in Table 5, none of the analyzed SNPs showed a significantly different genotype distribution between the TRD and non-TRD groups. We also tested the possible association between *PSMD9* and anxiety in 118 patients with and 972 patients without comorbid anxiety disorder. No difference in the rs1043307 genotype frequency distribution was observed between these two groups (*P=*0.74).

### mRNA expression analysis on fibroblasts

**Figure 1:**
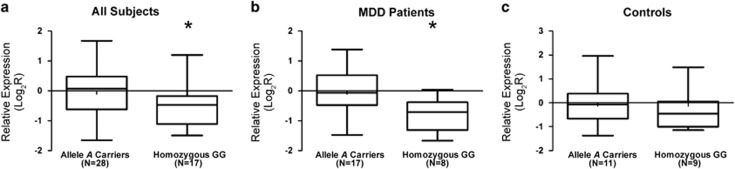
Relative expression mRNA levels of PSMD13 rs3817629. Box-plots showing the median, quartiles and extreme values of relative expression of *PSMD13* rs3817629 A allele carriers with respect to GG subjects. The bold lines represent median values, the box boundaries mark the 25th and 75th percentiles of each distribution, respectively. Whiskers, above and below the hinges, mark the largest and smallest observed values. (**a**) the analysis was performed in all subjects; (**b**) only in MDD group; (**c**) only in control group. '*' Indicates significant difference (*P*-value<0.05) computed by the Mann–Whitney *U* non-parametric test. MDD, major depressive disorder.

The analyses of the expression levels of *PSMD13* mRNA in fibroblasts from MDD subjects and controls revealed no significant differences between the two groups, as well as comparing TRD and non-TRD patients. Furthermore, no significant association with any examined socio-demographic or clinical variables was detected (data not shown). However, we found a trend toward a correlation between the *PSMD13* genotype and its mRNA levels (*H*=5.10; 2 degree of freedom, *P=*0.08). In particular, the homozygous GG carriers displayed lower expression levels than the A allele carriers (z=−2.20; *P=*0.03) (Figure 1a). However, after stratifying the subjects into the control and MDD patient groups, this downregulation was observed only in MDD patients (*z*=−2.40; *P=*0.02) (Figure 1b), whereas in control sample this association was not obtained (11A allele carriers, 9 GG; *z*=−0.57; *P=*0.60) (Figure 1c).

Regarding the expression of *PSMD9*, the analyses did not reveal any significant differences in its mRNA levels (data not shown).

### mRNA expression analysis on PBCs

The analyses of *PSMD13* mRNA levels in PBCs did not show any differences, both when we compared MDD and controls, as well as TRD and non-TRD patients. No association, neither with *PSMD13* polymorphism nor with any socio-demographic or clinical variables, was detected.

**Figure 2:**
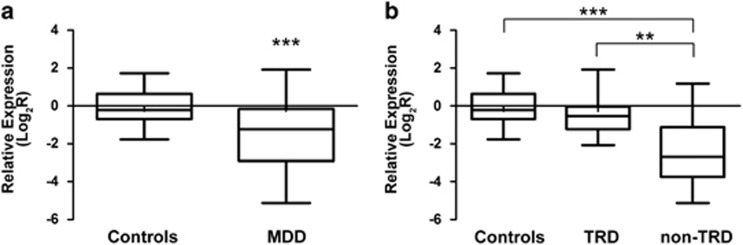
Relative expression mRNA levels of PSMD9 rs1043307. Box-plots showing the median, quartiles and extreme values of relative expression of *PSMD9* rs1043307 in (**a**) MDD patients and controls; (**b**) in healthy subjects, TRD and non-TRD. The bold lines represent median values, the box boundaries mark the 25th and 75th percentiles of each distribution, respectively. Whiskers, above and below the hinges, mark the largest and smallest observed values, respectively. '**' Indicates significant difference (*P*-value<0.01), whereas '***' indicates a *P*-value <0.001, computed by the Kruskal–Wallis and Mann–Whitney *U* non-parametric tests.

As concerned *PSMD9*, we observed a significant downregulation in MDD patients compared with healthy controls (*z*=−3.75; *P=*1.80 × 10^−4^) (Figure 2a). Pairwise comparisons indicated high differences between non-TRD patients group and both controls (*z*=−4.51; *P=*6.54 × 10^−6^) and TRD patients (*z*=−3.38; *P=*0.001), whereas, no significant difference was observed between the TRD patients and the healthy subjects (*z*=−1.70; *P=*0.09) (Figure 2b).

Our data also revealed significant correlations between *PSMD9* mRNA levels with the presence of psychotic symptoms (*r*=0.43; *P=*0.008), comorbidity with anxiety disorders (*r*=0.40; *P=*0.01) and smoke (*r*=−0.39; *P=*0.003). No significant correlation between mRNA levels with *PSMD9* rs1043307 SNP was observed.

## Discussion

In the present study, we found an association between the *PSMD13* rs3817629 G allele and TRD. In particular, homozygotic GG have about a twofold higher risk of developing MDD treatment resistance than A allele carriers. Our result supports the original observation of Wong *et al.*,^16^ who found that the A allele is associated with the antidepressant treatment response in a cohort of depressed patients.

LD analysis revealed that although rs3817629 does not appear to affect a binding site, it is in high LD with two SNPs, rs10794302 and rs10902110, which likely affect binding sites according to the Regulome database. In particular, Chip-seq analyses showed that rs10794302 maps to a locus corresponding to a GR-binding site. This result is intriguing because it has been observed that antidepressants exert their clinical effects via the modulation of the GR.^43^

Moreover, our expression analysis revealed that the homozygous GG genotype was associated with reduced transcription of *PSMD13* in MDD fibroblasts. This result suggests that rs3817629 is an expression quantitative trait locus in this tissue. Our finding is corroborated by a recent study that investigated the genetic variability in the regulation of gene expression in 10 regions of the human brain.^44^ The authors found that rs3817629 is a *cis*-expression quantitative trait locus in different areas of the brain and that this SNP is associated with the *PSMD13* transcriptional expression level (t3315549) in the entire brain (http://www.braineac.org/). Specifically, this result was primarily due to the differential expression observed in eight brain regions: the cerebellar cortex, the hippocampus, the inferior olivary nucleus, the occipital cortex, the putamen, the temporal cortex, the thalamus and the intralobular white matter. In all of these tissues, as in our fibroblasts, the homozygous GG genotype was associated with lower *PSMD13* expression providing further evidence that fibroblasts serve as a good peripheral model for the study of biomarkers of the central nervous system.^22, 45, 46^

This transcriptional finding combined with our genetic main result, that the homozygous GG subjects have about a twofold higher risk of developing MDD treatment resistance, might suggest that this SNP may represent a risk factor for TRD due to alteration of PSMD13 transcription. Unfortunately, the small sample size of our TRD and non-TRD groups for expression analysis, do not allow us to test differences in the mRNA levels between these two groups as concern the GG genotype.

Under inflammatory conditions mediated by IFN-γ, the activation of the mTOR pathway enhances protein translation and induces protein-damaging oxygen radical production; these events contribute to an increase in the synthesis of proteins that fail to fold correctly, referred to as defective ribosomal products and in proteasomal activity.^47, 48^ We suggest that subjects carrying the GG genotype and, thus, a low expression level of *PSMD13* may respond less effectively to conditions of augmented need for defective ribosomal product degradation, such as those caused by stress or an inflammatory response, which are observed in MDD. Therefore, such conditions could lead to more severe phenotypes and to worse drug treatment responses in MDD patients.

Our negative pharmacogenetic results for *PSMD9* did not confirm the previous finding reported by Wong *et al.*;^16^ however, a recent study has shown that the *PSMD9* missense SNP rs1043307 co-segregates with generalized anxiety disorder in type-2 diabetes families.^18^ Interestingly, we found a positive association between this SNP and the presence of anxiety disorders in comorbidity with MDD. Although our result did not remain significant after correction for multiple comparisons, taken together these data support the putative role of *PSMD9* as a risk factor for anxiety phenotypes.

Anxiety disorders co-occur in the same individual, either simultaneously or sequentially, with MDD (in ~40–50% of patients), and it has been widely demonstrated that anxiety impairs depression remission.^49^ Furthermore, several pieces of evidence led to the hypothesis that these two phenotypes have partially shared genetic bases.^49, 50, 51, 52^ This observation suggests that *PSMD9* variants may be indirectly implicated in the response through an involvement in one of the most important negative predictor of response like anxiety.

The likely involvement of *PSMD9* in MDD phenotypes was also suggested by our transcriptional data. In fact, mRNA expression analysis of PBCs revealed that *PSMD9* is significantly downregulated in MDD patients, specifically non-TRD patients, compared with controls. This result is in agreement with those observed in one of the largest genome-wide gene expression study in MDD.^19^ The results of single-gene association with MDD status, available on request from the National Institute of Mental Health (NIMH) Center for Collaborative Genomic Studies on Mental Disorders, (https://www.nimhgenetics.org/access_data_biomaterial.php), indicated that the mRNA expression levels of *PSMD9* gene were decreased in whole blood of MDD patients (nominal *P*-value=0.02).

However, our PBCs transcriptional levels are strongly associated to the presence of psychotic symptoms, the comorbidity with anxiety disorders and smoke. Thus, it is difficult to clarify if rather than being the cause of MDD, the alterations of *PSMD9* could probably be a consequence of the disease, since it is well-known that a depressive state has an adverse influence on lifestyle.

For both genes (*PSMD13* and *PSMD9*), we observed a discrepancy between PBCs and fibroblasts expression results. This could be explained by biases due to confounding factors that probably affect PBCs findings. Indeed, fibroblasts are maintained in cultures in a controlled, reproducible environment, and after several rounds of cell division they are minimally affected by confounding factors (for example, lifestyle or medication use) to which, in contrast, PBCs are exposed.^22, 35, 46, 53, 54^

The genetic analyses performed on the STAR*D cohort were not consistent with the results from our cohort or from Wong *et al.*^16^ These contrasting results could be explained by different reasons: (1) neither SNP was directly genotyped in the STAR*D cohort, but, albeit with high confidence, they were imputed, and therefore, the analysis was performed on predicted genotypes rather than on actual genotypes; (2) our samples and those of Wong *et al.*^16^ and the STAR*D cohort differed with respect to certain clinical characteristics. For instance, the STAR*D study included only outpatients with non-psychotic MDD, whereas both our sample than the Wong *et al.*^16^ one include MDD patients with psychotic symptoms. In addition, only ~11% of the patients in the selected STAR*D cohort presented anxiety disorders in comorbidity, whereas this percentage was 40% in our sample and in the study by Wong *et al.*^16, 55^ the patients showed an high severity of anxiety. As mentioned above, anxiety is a relevant negative predictor of the treatment response; moreover, in our cohort and in the STAR*D cohort we stratified in TRD and non-TRD patients, whereas Wong *et al.*^16^ stratified in responders and non-responders to desipramine or fluoxetine; and (3) the ethnicity in the STAR*D cohort is more heterogeneous than in the cohort of Wong *et al.*^16^ and in ours. This could represent an additional confounding factor.

In conclusion, despite the limitations of this study, our findings provide convergent evidence that the dysregulation of the ubiquitin–proteasome system is involved in MDD response treatment mechanisms. Independent replications are needed to better understand the role of this system in psychiatric disorders.

## Figures and Tables

**Table 1:** Demographic and clinical characteristics of both the control and MDD patient groups, as well as the non-TRD and TRD patient subgroups

*Characteristics*	*Controls (*N=*467)*	*MDD (*N=*621)*	P*-value*
Age (years), mean (s.d.)	46.9 (16.3)	56.2 (13.7)	<0.001
Gender (F), *n* (%)	260 (55.7)	422 (68.0)	<0.001
Education (years), mean (s.d.)	12.7 (4.7)	9.2 (4.0)	<0.001
	*TRD (*N=*390)*	*Non-TRD (*N=*231)*	
Age (years), mean (s.d.)	56.8 (13.4)	55.2 (14.1)	0.16
Gender (F), *n* (%)	260 (66.7)	162 (70.1)	0.37
Education (years), mean (s.d.)	8.9 (3.9)	9.8 (4.1)	0.007
% Of recurrent MDD	90.5	70.1	<0.001
% Of severe vs moderate MDD	87.9	45.0	<0.001
% Psychotic symptoms	35.6	3.5	<0.001
% Comorbidity with personality disorders	26.7	15.2	0.001
% Comorbidity with anxiety disorders	43.6	38.1	0.18
% Comorbidity with alcohol abuse	3.3	4.8	0.37

Abbreviations: F, female; MDD, major depressive disorder; PBC, peripheral blood cell; TRD, treatment-resistant depression.

**Table 2:** Demographic and clinical characteristics of both the control and MDD patient groups, as well as the TRD and non-TRD subgroups, from which fibroblast and PBC samples were collected

	*Fibroblasts*			*PBCs*		
*Characteristics*	*Controls (*N=*21)*	*MDD (*N=*27)*	P*-value*	*Controls (*N=*22)*	*MDD (*N=*37)*	P*-value*
Age (years), mean (s.d.)	46.6 (11.9)	51.2 (12.8)	0.66	32.1 (5.1)	49.9 (12.4)	<0.001
Gender (% F)	47.6	66.7	0.18	50.0	73.0	0.08
Education (years), mean (s.d.)	13.7 (5.8)	8.6 (3.5)	0.001	16.8 (4.1)	10.6 (4.6)	<0.001
BMI	24.8 (2.5)	26.1 (6.0)	0.41	23.3 (2.6)	25.3 (4.3)	0.07
% Smokers	4.8	40.9	0.005	22.7	50.0	0.04
	*TRD (*N=*10)*	*Non-TRD (*N=*17)*		*TRD (*N=*17)*	*Non-TRD (*N=*20)*	
Age (years), mean (s.d.)	48.6 (9.7)	52.7 (14.4)	0.43	52.3 (11.9)	47.7 (12.7)	0.25
Gender (% F)	64.7	70.0	0.78	64.7	80.0	0.30
Education (years), mean (s.d.)	8.7 (4.2)	8.6 (3.2)	0.94	10.8 (5.9)	10.4 (3.2)	0.78
BMI	28.5 (6.1)	25.2 (6.0)	0.36	25.2 (3.6)	25.5 (5.0)	0.85
% Smokers	33.3	46.2	0.55	35.3	64.7	0.09
% Of recurrent MDD	100.0	82.4	0.16	100.0	45.0	<0.001
% Of severe vs moderate MDD	50.0	47.1	0.88	88.2	45.0	0.006
% Psychotic symptoms	0.0	5.9	0.43	70.6	0.0	<0.001
% Comorbidity with personality disorders	40.0	35.3	0.81	76.5	25.0	0.002
% Comorbidity with anxiety disorders	40.0	29.4	0.57	64.7	25.0	0.02
% Comorbidity with alcohol abuse	20.0	0.0	0.06	5.9	0.0	0.27

Abbreviations: BMI, body mass index; F, female; MDD, major depressive disorder; PBC, peripheral blood cell; TRD, treatment-resistant depression.

**Table 3:** Logistic regression results for the genotype frequency distributions between the CTRL and the MDD patients

*SNP*	*Major homozygous frequency*	*Heterozygous frequency*	*Minor homozygous frequency*	*Regression* P*-value additive model*^a^	*PGC* P*-value*
PSMA7 rs2057168	AA	AG	GG		
CTRL	296 (0.64)	148 (0.32)	21 (0.04)	0.59	0.48
MDD	399 (0.65)	186 (0.30)	27 (0.04)		

PSMD9 rs1043307	AA	AG	GG		
CTRL	207 (0.44)	219 (0.47)	40 (0.09)	0.23	0.68
MDD	303 (0.49)	255 (0.41)	59 (0.10)		

PSMD13 rs3817629	GG	AG	AA		
CTRL	224 (0.48)	202 (0.44)	37 (0.08)	0.41	Not genotyped
MDD	311 (0.51)	261 (0.42)	42 (0.07)		

Abbreviations: CTRL, control; MDD, major depressive disorder; PGC, Psychiatric Genomics Consortium; SNP, single nucleotide polymorphism.

a Gender, age and education were considered as covariates in the model.

**Table 4:** Logistic regression results for the genotype frequency distributions between the non-TRD and TRD patients

*SNP*	*Major homozygous frequency*	*Heterozygous frequency*	*Minor homozygous frequency*	P*-value*^a^ *additive model*	P*-value*^a^ *dominant model*	P*-value*^a^ *recessive model*
PSMA7 rs2057168	AA	AG	GG			
Non-TRD	143 (0.62)	77 (0.33)	10 (0.04)	0.35	0.22	0.79
TRD	256 (0.67)	109 (0.29)	17 (0.04)			

PSMD9 rs1043307	AA	AG	GG			
Non-TRD	107 (0.46)	101 (0.44)	21 (0.09)	0.50	0.33	0.90
TRD	196 (0.51)	154 (0.40)	38 (0.10)			

*PSMD13* rs3817629	GG	AG	AA			
Non-TRD	94 (0.42)	115 (0.51)	16 (0.07)	**0.004**	**0.0005**	0.87
TRD	217 (0.56)	146 (0.38)	26 (0.07)			

Abbreviations: SNP, single nucleotide polymorphism; TRD, treatment-resistant depression.

a Education was considered as a covariate in the model. Bold numbers indicate significant P-values (<0.05).

**Table 5:** Logistic regression results for the genotype frequency distributions between the non-TRD and TRD patients in the STAR*D cohort

*Gene*	*SNP tested*	P*-value additive model*^a^^*,*^^b^	*Note*
*PSMA7*	rs1535669	0.76	LD *r*^2^=0.95 with rs2057168
*PSMD9*	rs1043307	0.30	Imputed with *r*^2^=0.89
*PSMD13*	rs3817629	0.98	Imputed with *r*^2^=0.94

Abbreviations: LD, linkage disequilibrium; PCA, principal component analysis; SNP, single nucleotide polymorphism.

a The first three PCAs were considered as covariates in the analysis.

b For the imputed SNPs, the B allelic dosage was used as independent variables in the regression.
