# Supplementary material for: Proteasome system dysregulation and treatment resistance mechanisms in major depressive disorder
Source: Transl Psychiatry. 2015 Dec 1;5(12):e687–. doi: 10.1038/tp.2015.180 (PMC5068581; doi:10.1038/tp.2015.180)
Supplement: Supplementary Figure 1 [file tp2015180x1.doc]

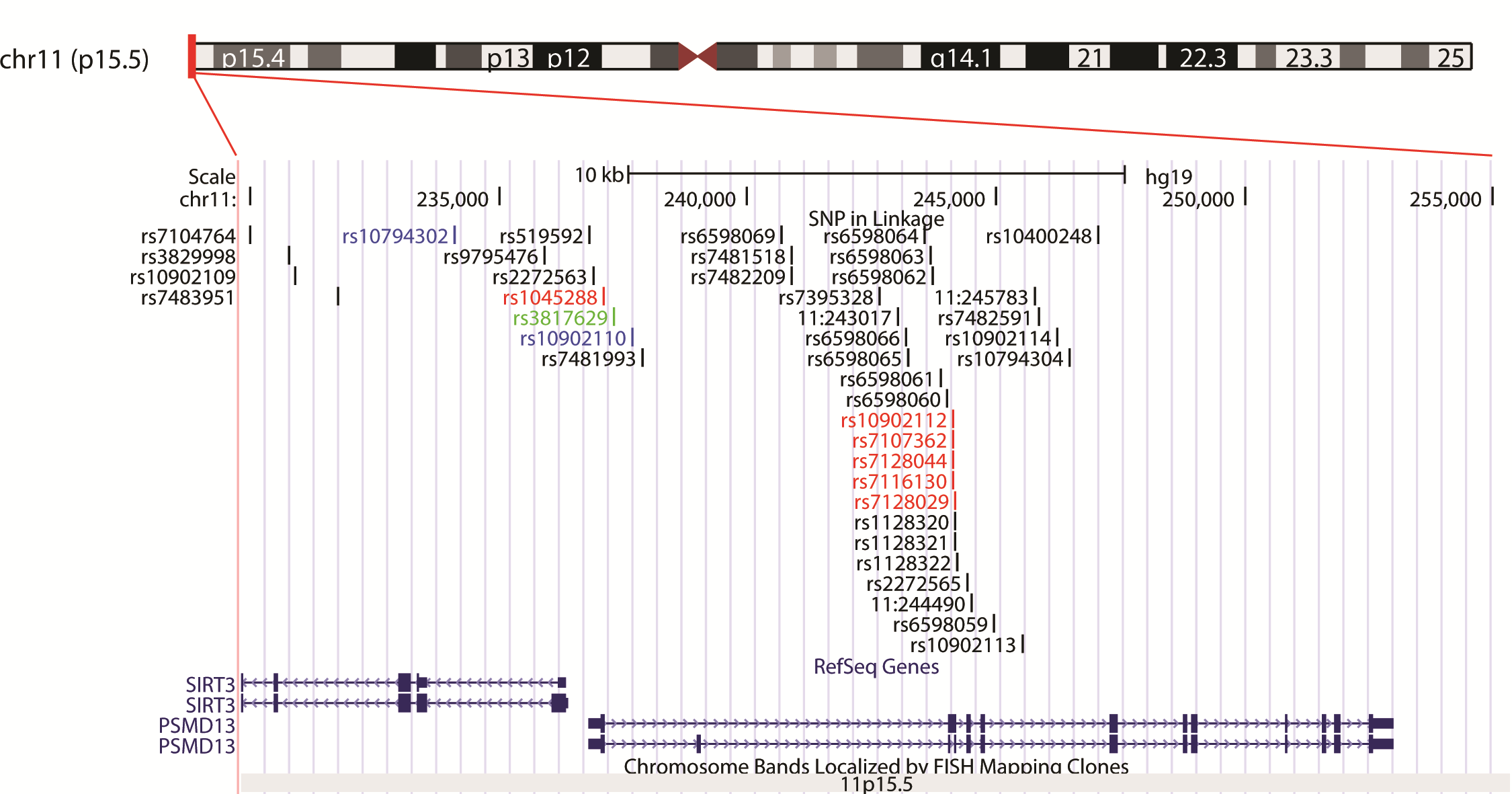


Supplementary Figure 1. Genome Browser visualization of the SNPs in LD with rs3817629. rs3817629 is depicted in green. Nonsynonymous SNPs are in red and the two SNPs with a Regulome score of 2b are reported in blue.
